# Gallic Acid and Quercetin as Intelligent and Active Ingredients in Poly(vinyl alcohol) Films for Food Packaging

**DOI:** 10.3390/polym11121999

**Published:** 2019-12-03

**Authors:** Francesca Luzi, Elisa Pannucci, Luca Santi, José Maria Kenny, Luigi Torre, Roberta Bernini, Debora Puglia

**Affiliations:** 1 Civil and Environmental Engineering Department, University of Perugia, Strada di Pentima 4, 05100 Terni, Italy; jose.kenny@unipg.it (J.M.K.); luigi.torre@unipg.it (L.T.); debora.puglia@unipg.it (D.P.); 2 Department of Agriculture and Forest Sciences (DAFNE), University of Tuscia, Via S. Camillo De Lellis, 01100 Viterbo, Italy; e.pannucci@unitus.it (E.P.); luca.santi@unitus.it (L.S.); roberta.bernini@unitus.it (R.B.)

**Keywords:** poly(vinyl alcohol) (PVA), gallic acid, quercetin, specific migration, antioxidant properties

## Abstract

Gallic acid (GA) and quercetin (QC) were used as active ingredients in poly(vinyl alcohol) (PVA) film formulations obtained by solvent casting process. The effect of two different percentages (5 and 10 % wt.) on morphological behavior, thermal stability, optical, mechanical, and release properties of PVA were investigated, while migration with food stimulants and antioxidant properties were tested taking into account the final application as food packaging systems. The results showed how different dispersability in PVA water solutions gave different results in term of deformability (mean value of ε _PVA/5GA_ = 280% and ε _PVA/5QC_ = 255%, with 190% for neat PVA), comparable values for antioxidant activity at the high contents (Radical Scavenging Activity, RSA(%) _PVA/10GA_ = 95 and RSA(%) _PVA/10QC_ = 91) and different coloring attitude of the polymeric films. It was proved that GA, even if it represents the best antioxidant ingredient to be used with PVA and can be easily dispersed in water, it gives more rigid films in comparison to QC, that indeed was more efficient in tuning the deformability of the PVA films, due the presence of sole hydroxyl groups carrying agent. The deviation of the film coloring towards greenish tones for GA films and redness for QC films after 7 and within 21 days in the simulated conditions confirmed the possibility of using easy processable PVA films as active and intelligent films in food packaging.

## 1. Introduction

**Figure 1:**
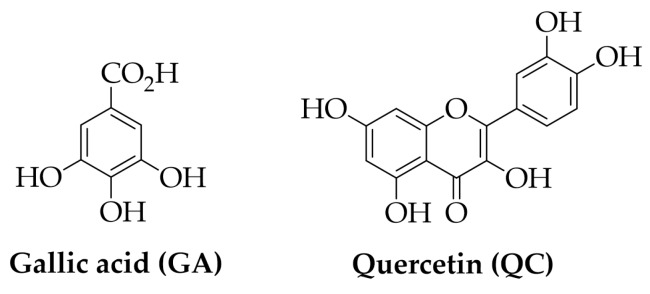
Chemical structure of gallic acid (GA) and quercetin (QC).

In the last few years, the use of pure phenolic compounds and phenolic extracts in active polymeric packaging has attracted a particular interest, since these compounds show potent antimicrobial and antioxidant activity in food systems and their intake can make a contribution to human health. Being the phenolic compounds able to interact with the environment and the product to extend shelf life, their addition to the package reduces the need to use synthetic antioxidants in the plastic, limiting the risk of potential toxicity by migration, and protects at the same time the package content. Equally, the extra protection of the food provided by the slow release of antioxidants from the package can make it possible to reduce the direct addition of chemicals to the food. Polyphenols belong to a wide category of plant derived natural compounds exhibiting many biological properties [1], including anticancer [2,3], anti-inflammatory [4,5], antimicrobial and antioxidant activity [6]. Among these compounds, gallic acid (3,4,5-trihydroxybenzoic acid, GA) and quercetin (3,3′,4′,5,7-pentahydroxyflavone, QC) are two potent antioxidants, due both to their redox properties and structural features (Figure 1), that impart high antioxidant activity: the three hydroxyl groups for GA [7] and the four hydroxyl groups on the A and B ring in combination with the 2,3-double bond, the 4-keto group and the 3-hydroxyl group in the C ring for QC [8]. Both GA and QC are found in the plant kingdom and in plant derived food, and beverages [1]. GA is present in nuts, green tea, and red wine, but also in oak bark and gallnuts as catechin derivatives and hydrolyzable tannins [9]. QC is abundant in onions, apples, berries, cherries, broccoli, red grapevines, and tea. Both compounds are commonly used as additives in cosmetics and food to prevent the oxidative processes responsible for product deterioration [10,11].

Limiting the analysis to their use in poly(vinyl alcohol) (PVA) based films, the literature reports the incorporation of tannic acid [10], hydroxytyrosol and its derivatives [11,12,13], tea plant [14,15,16] and rosemary extracts, to produce antioxidant films potentially useful to increase the shelf life of food [17,18].

The same approach was used in PVA blends and nanocomposites, as in the case reported by Wu et al. [19] and Matos de Carvalho et al. [20], where the authors respectively demonstrated that PVA–starch films impregnated with 500–1000 ppm of catechins and PVA cast film with solid lipid nanoparticles entrapping α-tocopherol showed relevant antioxidant capacity and controlled release of the active ingredients, confirming the possibility of their use in food preservation.

It is also well known in the literature that dyes containing phenolic or conjugated substances, are useful for colorimetric determination of pH in an intelligent manner. Thus, presence of polyphenols in polymeric films can act as pH indicators and this use has been extensively investigated. In the specific case of PVA based films, Ma et al. [21] developed a pH indicator which consisted of polyvinyl alcohol (PVA)-chitosan nanoparticles and mulberry extract and they found that change in color indicator from red to green was correlated with the presence of volatile nitrogenous compounds, which is characteristic of fish spoilage. Anthocyanins that were extracted from roselle immobilized onto starch-PVA-based film were also proposed by Zhai et al. [22] as visual colorimetric film for volatile nitrogenous compounds released in fish spoilage.

In addition, Liu et al. [23] developed an intelligent starch/poly-vinyl alcohol (PVA) capable of monitoring pH changes and inhibiting undesired microbial growth in pasteurized milk. The results suggested that the intelligent films reported here show good capability for both alerting and inhibiting food spoilage. A recent example of QC loaded PVA can be found in He at al. [24], where the authors prepared, via physically mixing QC aggregation-induced emission luminogens (AIEgens) as fluorescence sensors for detecting biogenic amines formed during the spoilage of seafood. Again, Ma et al. [25] prepared pH sensing film from tara gum-cellulose nanocrystal incorporated with natural dye (grape skin extract) to evaluate the pH changes of the milk at ambient temperature for 48 h. During the test, the color of indicator clearly changed from bright red (acidic) to dark green (alkaline), which can be correlated with microbial contamination and pH decrease. The authors suggested that the developed pH sensing film could be used as a visible color indicator and changes in color of the film provide information to monitor the packaged food freshness.

With the same aim, in parallel with morphological, thermal, and mechanical behavior of produced films, the antioxidant activity of PVA containing GA and QC at two different percentage (5 and 10% wt.) before and after release in food simulated solutions and their change in color were here investigated. The variations in terms of color for films was then correlated to swelling behavior of polymeric films in presence of the active ingredients, having in mind to use the produced films as indicators for safe food packaging applications.

## 2. Materials and Methods

### 2.1. Materials

All chemicals, poly(vinyl alcohol) (average MW 124–146 kg mol^−1^, 99% hydrolyzed) including gallic acid, quercetin dehydrate, and solvents of analytical grade were purchased from Sigma-Aldrich® (Milan, Italy).

### 2.2. Preparation of PVA Based Formulations

PVA based formulations were prepared by solvent casting technique. The concentrations of active GA and QC were fixed at 5 and 10% wt., as previously reported [26]. Firstly, 0.5 g of PVA was dissolved in 10 mL of distilled water under magnetic stirring for 2 h at 80 °C. The polymeric solution was kept under magnetic stirring to reach room temperature (RT).

**Scheme 1:**
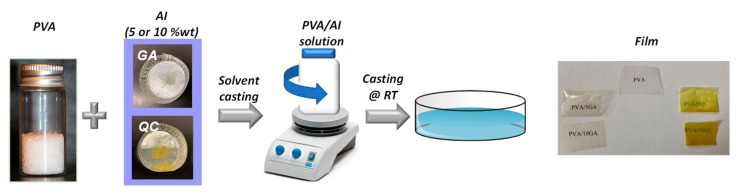
Procedure for the preparation of poly(vinyl alcohol) (PVA) formulations.

PVA based formulations were obtained mixing, under magnetic stirring, the polymeric solution with a specific amount of GA or QC, previously dispersed in distilled water (0.1 g of active ingredient in 10 mL of distilled water) for 1 h at RT. Uniform dispersion of QC or GA in aqueous solution was obtained applying a magnetic stirring and a sonication bath, both at RT for 1 h (Scheme 1).

The polymeric solutions of neat film and binary films were cast in a Teflon® mold and evaporated at RT. Films having thicknesses in the range 30–60 μm were obtained and equilibrated for 7 days at 53% RH (an oversaturated magnesium nitrate solution was used to control the RH) in desiccators, by using magnesium nitrate-6-hydrate oversaturated solution, after the processing and before the characterizations, in accordance to the literature [12,27].

### 2.3. Characterization of PVA Based Formulations

Field emission scanning electron microscopy (FESEM, Supra 25-Zeiss) was used to investigate morphologies of both GA and QC and fractured surface (surface obtained after the tensile test) morphology of PVA based formulations. The surfaces were gold sputtered in order to offer electric conductivity and the samples were observed using an accelerating voltage of 2.5 kV.

FTIR measurements for QC, GA, and PVA formulations were performed by using a Jasco FT-IR 615 spectrometer (Jasco Inc., Easton, MD, USA) in the 400–4000 cm^−1^ range in attenuated total reflection (ATR) mode.

Thermal characterization of active ingredients and PVA based formulations was carried out by both differential scanning calorimetric (DSC) and thermogravimetric analysis (TGA).

DSC (DSC-TA Instrument, Q200) measurements were performed in the temperature range from −25 to 240 °C, at a heating rate of 10 °C min^−1^. Three samples were used to characterize each material.

The glass transition temperature (*T_g_*), melting temperature (*T_m_*), crystallization temperature (*T_c_*), and relative enthalpies (Δ*H_m_* and Δ*H_c_*) were evaluated.

The crystallinity degree (melting crystallinity degree (*X_m_*) and crystallinity degree at cooling scan (*X_c_*)) was calculated according to the following Equation (1):

(1) $$\chi =\frac{1}{\left(1-m_{f}\right)}\left[\frac{\Delta H}{\Delta H_{0}}\right]\times 100$$

where Δ*H* is the enthalpy for melting or crystallization; Δ*H*_0_ is enthalpy of melting for a 100% crystalline PVA sample, taken as 161.6 J g^−1^ [28,29] and (1 − m_f_) is the weight fraction of PVA in the sample.

TGA (Seiko Exstar 6300) experiments were performed for each film from 30 to 600 °C at 10 °C min^−1^ under a nitrogen atmosphere (250 mL min^−1^) in order to evaluate the effect active ingredient (AI) addition on the thermal stability of PVA matrix.

The mechanical properties (tensile tests) of neat PVA and PVA/AI based systems were performed on rectangular probes as indicated in the EN ISO 527-5 standard, with a crosshead speed 5 mm min^−1^, a load cell 500 N by using a digital Lloyd testing machine (Lloyd Instrument LR 30K Segens worth West, Foreham, UK).

Average tensile strength (σ_B_), elongation at break (ε_B_), and Young’s modulus (E) were calculated from the resulting stress-strain curves. The measurements were done at room temperature and at least five samples for each formulation were tested.

The overall migration analysis of PVA based formulations was done in triplicate in simulant D (50% (*v/v*) ethanol/water solution) according to current legislation Commission Regulation (EU) No 10/2011. The analysis was performed to simulate the behavior of polymeric based films in contact with food simulant. Rectangular strips of 10 cm^2^ in 10 mL of food simulant were used (Commission Regulation, EU 10/2011). The samples were kept in a controlled chamber at 40 °C, removed after 10 days according to EN-1186 standard, and the simulant was evaporated in dishes and dried at 105 °C for 2 h in an oven. The residues were weighed with an analytical balance (Sartorius ATILON) with ±0.01 mg precision and the migration values in mg kg^−1^ of the/each simulant were determined.

Specific migration tests were performed into ethanol/water 50% (*v/v*) solution as food simulant according to European Standard EN 13130-2005 (UNE-EN 13130–1: 2005) and European Commission Regulation 10/2011 (Commission Regulation, EU 10/2011). Double-sided, total immersion migration tests were carried out with 12 cm^2^ of films and 20 mL of simulant (area-to-volume ratio around 6 dm^2^ L^−1^) in triplicate at 40 °C in an oven (J. P. Selecta, Barcelona, Spain). Samples were taken at 1, 3, 7, 10, 21 days in triplicate; a blank test for the simulant was also included. After the migration tests, each solution was recovered and stored at −4 °C before analysis.

The color variations of PVA films were investigated by means of a spectrophotometer (CM-2300d Konica Minolta, Japan). Data were acquired by using the SCI 10/D65 method whereas CIELAB color variables, as defined by the Commission Internationale de 1′Éclairage (CIE 1995), were used. Film specimens were placed on a white standard plate and L*, a*, and b*parameters were determined. L* value ranges from 0 (black) to 100 (white); a* value ranges from −80 (green) to 100 (red); and b* value ranges from −80 (blue) to 70 (yellow). Samples were analyzed in triplicate, and three measurements were taken at random locations on each of the studied films. The total color difference ΔE* and gloss values were calculated as indicated in Equation (2):

(2) $$\Delta E^{*}=\sqrt{{\left(\Delta L^{*}\right)}^{2}+{\left(\Delta a^{*}\right)}^{2}+{\left(\Delta b^{*}\right)}^{2}}$$

### 2.4. GA and QC Release Studies in Food Simulant (Specific Migration)

GA and QC released by PVA based formulations in food simulant for every contact time (1, 3, 7, 10, and 21 days) were analyzed by UV-2600 spectrophotometer (Shimadzu, Kyoto, Japan). Absorbance values were measured at 270 nm and at 375 nm for GA and QC, respectively. Standard curves for GA (range 4.0–20 μg mL^−1^) and QC (range 2.0–14 μg mL^−1^) have been used. Samples were analyzed in triplicate and results are the mean ± standard deviation (SD) and expressed as μg of antioxidant per mL of food simulant.

### 2.5. Antioxidant Activity of Films and Food Simulant Solutions

#### 2.5.1. Antioxidant Activity of Films

Radical scavenging activity of PVA based films at initial and after the contact with food simulant (simulant D, 50 *v/v*% of ethanol) at different times (1, 3, 7, 10, and 21 days) was determined by using a spectroscopic method according to the procedure proposed in literature by Byun et al. [30]. The different PVA systems (0.1 g) were cut into small pieces and immersed in 2 mL of methanol for 24 h at RT. An aliquot of methanol extract (1 mL) was mixed with 1 mL of 2,2-diphenyl-1-picryl-hydrazyl-hydrate (DPPH) in methanol (50 mg L^−1^). The mixture was allowed to stand at RT in the dark for 60 min. The absorbance was measured at 517 nm using a UV spectrometer (Lambda 35). The DPPH mixture solution of methanol extracted from neat PVA was used as control. DPPH radical scavenging activity (RSA) was measured according to the Equation (3):

(3) $$RSA\left(\%\right)=\frac{A_{Control}-A_{Sample}}{A_{Control}}\times 100$$

where *A*_sample_ is the absorbance of sample and *A*_control_ is the absorbance of the control.

#### 2.5.2. Antioxidant Activity of Food Simulant Solutions

The antioxidant activity of the food simulant solutions was determined using the DPPH assay according to the literature already reported by us [11,12]. Briefly, 1.5 mL of DPPH solution (50 mg L^−1^) was mixed with 1.0 mL of samples and the optical density was recorded at 517 nm after 60 min of reaction in the dark. The DPPH radical scavenging activity was expressed as RSA% according to the Equation (3). The DPPH mixture solution of simulant from neat PVA films was used as control.

### 2.6. Statistical Analysis

The experimental results were expressed as mean ±SD of three replicates. Statistical differences were calculated using one-way analysis of variance (ANOVA), followed by Tukey’s honest significant difference (HSD) post hoc test.

## 3. Results and Discussion

### 3.1. GA and QC Characterization

**Figure 2:**
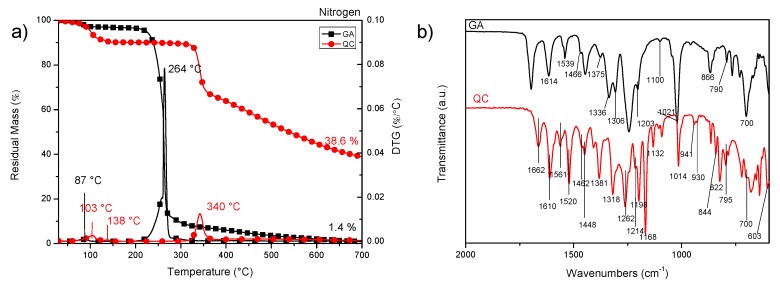
(**a**) TG and DTG curves and (**b**) FTIR spectra for gallic acid (GA) and quercetin (QC) powders.

The thermal stability of QC and GA in inert atmosphere was determined by TGA tests, with a run from 30 to 700 °C under nitrogen at 10 °C/min. Figure 2a shows TG/DTG profiles for GA and QC. In the case of QC, that it is proved to be highly hygroscopic, the first loss was observed at 103 °C, due to the loss of H_2_O molecules from the crystal lattice of its hydrate form [31,32]. The dehydration is complete at 160 °C, no further mass loss was observed until 300 °C, which marks the onset of its thermal degradation [33]. After that, a sharp exothermic peak was observed in QC at 340 °C, due to decomposition of organic matter: we found that a significant amount of complex (38.6%) remains after heating the polymer to 700 °C, and this is attributed to the formation of new catechol-catechol bonds [34]. In the case of GA, the first low intensity degradation peak centered at 87 °C is due to the degradation of water and volatile compounds with low molecular weight, while a main peak centered at ca. 264 °C, that corresponds to the main chain scission, was detected [35]. Finally, minor and progressive degradation phenomena of lower intensity were observed at temperatures above 400 °C, which are related to the residual decomposition of GA at high temperature [36].

The FTIR spectra for QC and GA are shown in Figure 2b. In the case of QC, OH groups stretching were detected at 3403, 3321 (not shown), 1448, and 1014 cm^−1^ (the large presence of OH groups accounts for the solubility of the polymer in mix solvents such as ethanol/water), whereas OH bending of the phenol function was detectable at 1381 cm^−1^. The C=O aryl stretching absorption was evident at 1662 cm^−1^. Aromatic ring stretch bands for C=C were detected at 1610, 1561, and 1520 cm^−1^. The signal for ν(C–O) can be found at 1462 cm^−1^ [37], other bands associated with the vibrations of C–O bonds appear also at 1132 cm^−1^ (C–OH stretching). The in-plane bending band of C–H in aromatic hydrocarbon was detectable at 1318 cm^−1^, and out-of-plane bending bands were evident at 930, 822, 678, and 603 cm^−1^. Bands at 1262, 1198, and 1168 cm^−1^ are related, respectively, to the C–O stretching in the aryl ether ring, the C–O stretching in phenol, and the C–CO–C stretch and bending in ketone [38,39], other signals for C–OH deformation and –C–OH stretch vibration were found at 1214 cm^−1^ and 1091 cm^−1^. Minor signals attributable to substituted benzene can be found at 941, 864, 795, and 700 cm^–1^ [40].

FTIR spectrum for GA powder exhibits characteristic peaks at 3482 cm^−1^ and 3281 cm^−1^, which are due to stretching of the O–H groups (not shown), a sharp absorption band of carboxylic group –COOH at 1695 cm^−1^, absorption bands of –OH at 866 cm^−1^, as well as –C–OH at 1320 cm^−1^, 1375 cm^−1^ (Phenol (or) tertiary alcohol, OH bend), 1244 cm^−1^ (C–O stretch) and peak at 766, 1021, 1100, and 1614 cm^−1^, a C=C stretching vibration can be observed at 1539 cm^−1^ [41]. The peaks located at 1614, 1539, 1446 cm^−1^ represented the aromatic ring C=C stretching vibrations [42]. There are several peaks in 1300–1000 cm^−1^ region (1336, 1306, 1203 cm^−1^) that could be assigned to the stretching vibration of C–O bond and bending vibration of O–H [43]. Minor signals related to substituted benzene can be found at 985, 790, and 700 cm^−1^.

**Figure 3:**
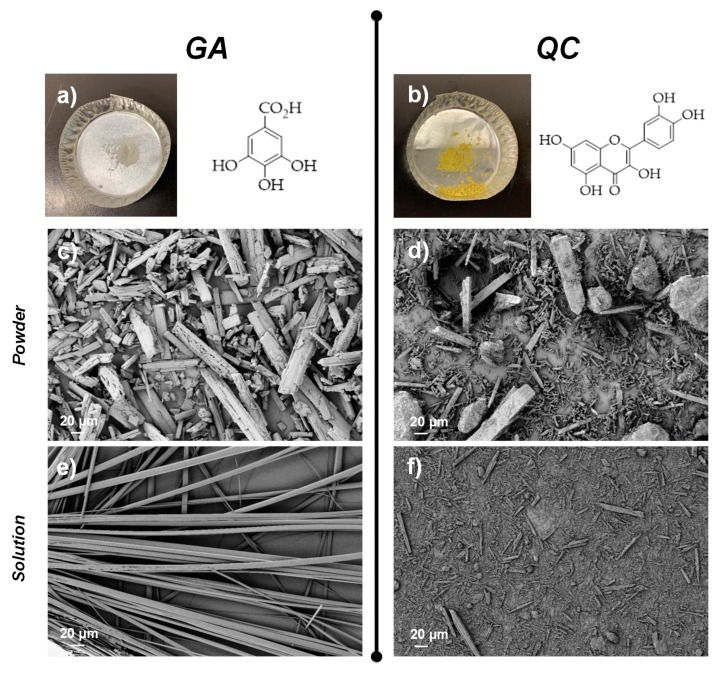
Visual aspect and chemical formula for GA (**a**), FESEM image of powder before (**c**) and after (**e**) dispersion in water prior to sonication; visual aspect and chemical formula for QC (**b**), FESEM image of powder before (**d**) and after (**f**) dispersion in water.

GA powder (appearance and chemical formula in Figure 3a) was characterized by crystals of small size and regular shape with an apparently smooth surface [44] (Figure 3c) while, after water dispersion/drying, appeared individualized with a fibrous structure, reduced diameters, and increased lengths. In details, FESEM image (Figure 3e) shows that GA was organized as long (10–100 µm) filaments with diameter of 500–550 nm.

In the case of poorly water soluble QC (appearance and chemical formula in Figure 3b), it has been found that, in its powdered form, it consists of irregular rod structures [45,46] (Figure 3d) while, after dispersion in water, reduced particle size was noted (Figure 3f): particle size uniformity was obtained and lack of larger needle-type crystalline structures was found [47,48], in analogy with a previous study where QC morphology in relationship to the interaction with water under room temperature conditions was analyzed [49].

### 3.2. Characterization of PVA Based Formulations

#### 3.2.1. Morphological Properties

**Figure 4:**
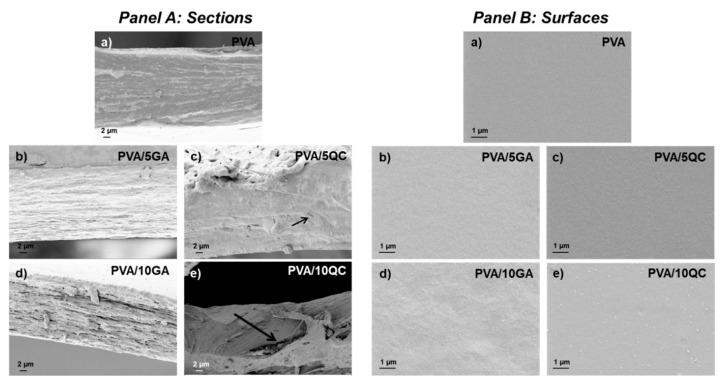
FESEM microstructure of fractured cross-sections (Panel A) and surfaces (Panel B) of PVA (**a**), PVA/5GA (**b**), PVA/5QC (**c**) PVA/10GA (**d**), and PVA/10QC (**e**) based formulations.

Figure 4, (Panel A) shows the microstructure of the fractured sections of PVA based formulations. PVA film is characterized by a homogeneous, uniform, and smooth fractured surface (Figure 4a) [29,50]. This behavior underlines the good processability and film-forming ability of PVA during solvent casting process. The addition of GA and QC affects the homogeneity and the uniformity of PVA films at both concentrations (5 and 10 wt. %). GA in polymer matrix processed by solvent casting results well dispersed, due to its high hydrophilicity and solubility in water solution [51]. The PVA/GA based films show a porous stretched fractured surface, due to the GA rearrangement during the evaporation phenomenon (Figure 4b,d). The FESEM images of PVA/QC based films (Figure 4c,e) show the presence of QC not properly solubilized in aqueous solution (see the arrows). As already observed by FESEM analysis for QC, after dispersion in water solution reduced particle size was noted (Figure 4d) [47]. In Figure 4, Panel B, images for surfaces of the same samples are reported: the results confirmed the presence of a flat and uniform morphology for the different materials, that was substantially unaffected by the presence of the active ingredients (only a limited roughness was found in the case of QC containing films).

#### 3.2.2. Thermal Analysis

Thermal properties of PVA and PVA based films were evaluated by differential scanning calorimetry (DSC) and thermogravimetric analysis (TGA) under nitrogen atmosphere, with the goal of assessing the effect of both GA and QC at different weight contents on thermal stability of PVA matrix.

Table 1 summarizes the values of parameters related to glass transition, crystallization and melting phenomena of PVA and PVA based films, measured during the cooling and the second heating scans. In the cooling scan, a decrease in the *T*_g_ with the increase of active ingredients amount was noted, indicating that the mobility of the PVA chains, probably due to electrostatic interactions between the GA (or QC) and the PVA, diminished [52]. During the cooling, when the content was increased from 0 to 5% for both GA and QC, the crystallization enthalpy increased, manifesting that the crystallinity also increased. However, PVA/10GA and PVA/10QC showed a reduced crystallinity value: apparently, higher contents of active ingredients can hinder the chains packing and inhibit crystals growth, as already observed by Peng et al. [53]. Moreover, the analogous trend was found for the crystallization temperature Tc, that gradually decreased with the addition of GA and QC from 5 to 10% wt. [10].

During the second heating scan, neat PVA glass transition temperature occurred at 77 °C. With the addition of both GA and QC at the two different amounts, *T*_g_ gradually increased to a mean value of 85 °C when 10% wt. of both active ingredients was incorporated, whereas a notable enhancement of T_m_ values during the second heating scan, due to reduced mobility of PVA chains, was also registered [15]. In addition, PVA/GA and PVA/QC films exhibited narrower endothermic peaks than PVA alone (data not shown). Zhu et al. also observed that when polyphenol catechins are mixed with poly(ε-caprolactone) or poly(3-hydroxypropionate), only one T_g_ exists for all component ranges, and this value increases as the content of polyphenol in the system increases [54].

**Figure 5:**
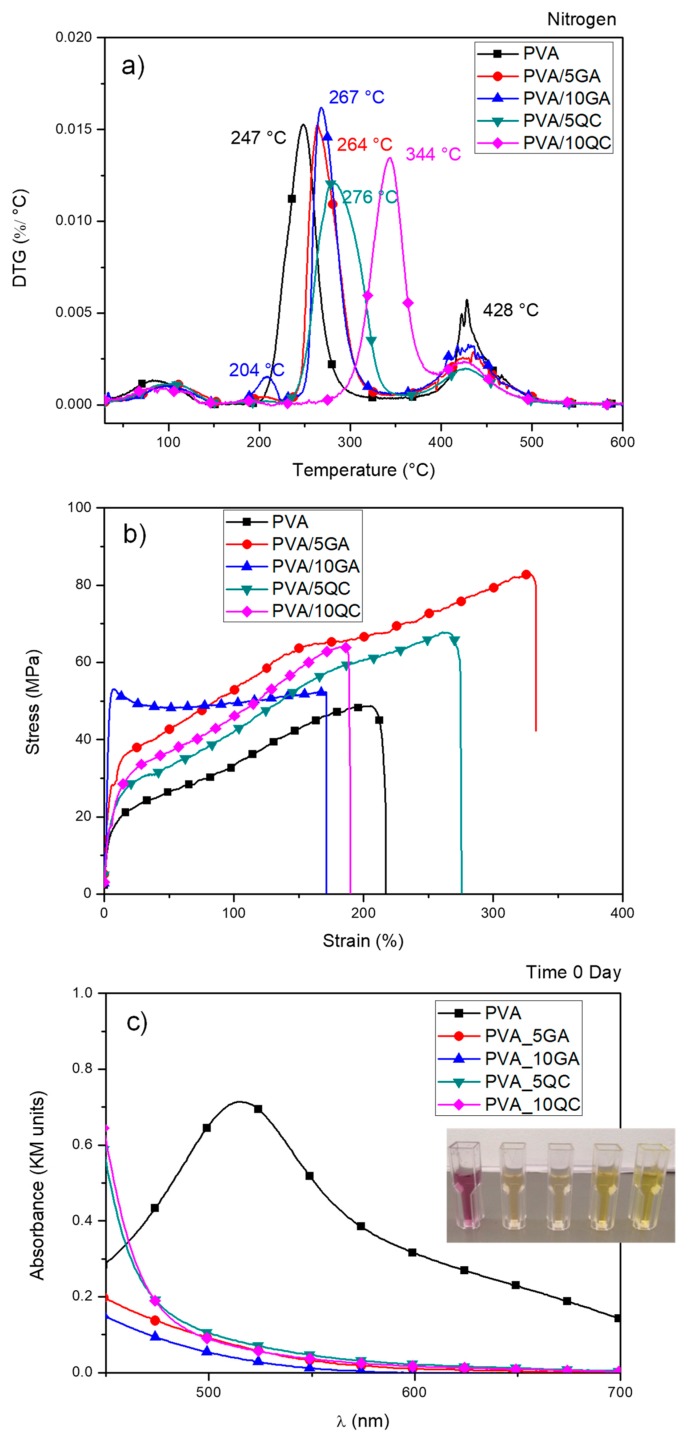
Mass loss and derivative weight loss curves (nitrogen) for PVA/GA and PVA/QC formulations at 5 and 10% wt. (**a**); Stress-strain curves from tensile tests for PVA/GA and PVA/QC (**b**); reduction of stable free radical DPPH (Radical scavenging activity, RSA) for PVA/GA and PVA/QC based formulations (**c**).

The effect of QC and GA on degradative behavior of PVA matrix was also investigated by TGA, mass loss and derivative weight loss curves are reported in Figure 5a. All the studied formulations were characterized by the presence of a multi-step degradation behavior: the first weight loss, at low temperature centered at around 80–110 °C, corresponds to the removal of weakly bound water or dehydration. The second and the third degradation temperatures correspond to the degradation of neat PVA [55]. In particular, the second/main degradation step of PVA film corresponds to the chain scissoring, with removal of residual acetate groups, due to the incomplete hydrolysis of PVA that remains in the chains, while the third degradation step is due to the cyclization reaction and continual elimination of residual acetate groups [13].

GA containing films displayed enhanced T_max_ and decrease in the rate of degradation (indicated by the broadening of DTG curve), with the shift of the second degradation peak from 247 °C for neat PVA to 264 and 267 °C, respectively for PVA/5GA and PVA/10GA. It is considered that interactions between the terminal –OH groups of the biopolymer chain with –OH, –CO, and/or –COOH chemical groups of the GA components via hydrogen bonding are responsible for the observed thermal stability improvement [56,57]. QC containing films showed prominent effect on the thermal properties with huge improvement in the *T*_max_ values, with the shift of the second degradation peak to 276 and 344 °C, respectively for PVA/5QC and PVA/10QC, confirming the observation of Dhand et al. [58], where it was found that strong covalent or non-covalent interactions between the −OH groups of PVA with −OH moieties of heteroaromatic catechol structures conferred a thermal shift, that was more evident in the case of complex polyphenols.

#### 3.2.3. Mechanical Response

The mechanical response of the active ingredients based PVA films was here investigated taking into account the final application in the industrial sector for which the mechanical characteristics are critical issues. The performed tensile tests permitted to evaluate the influence of GA and QC on the PVA mechanical properties, experimental results are reported in Figure 5b and Table 2.

The elongation at break of PVA neat film was measured at 195 ± 45%, underlining the ductile nature of the selected polymer matrix, as already previously observed in the literature [12]. The addition of different amounts of GA and QC to PVA induced a modification in the plastic response of PVA matrix. In particular, the deformation at break for PVA/5GA increased to about 280% as a result of a plasticizing effect [59], while the value was maintained similar to unfilled system by adding 10% wt. of GA in the PVA matrix. It is reasonable to argue that the presence of phenolic compounds having hydrophilic properties might have contributed to increase the inter-chain interactions, reducing the flexibility of the PVA films [57,60].

In comparison with neat PVA, a limited decrease of strength values was also observed, that can be explained by the change of intramolecular bonding with the addition of GA and QC. PVA/5QC films showed tensile strength values higher than PVA/5GC, and this result can be related to the inter-molecular interactions between hydrophilic groups in PVA and polyhydroxyl groups of quercetin [61]. On the other hand, tensile strength of PVA/10QC was weaker than that of PVA. This was because the incorporation of excessive amount of hydrophobic quercetin made the inner structure of PVA film become discontinue [62]. In analogy with results from Yoon et al. [63], that reported the variation in flexibility and strength of PVA films containing additives having both hydroxyl and carboxyl groups (glycerol and succinic acid) and glycerol (only hydroxyl groups), in our case we observed that, when GA having both hydroxyl and carboxyl groups is added to PVA films, more rigid films were found than those with only hydroxyl group-containing agent (QC).

#### 3.2.4. Overall Migration, Antioxidant Activity of PVA Formulations and Simulant Solutions at Different Times

Overall migration test is used to determine the limit value at which the substances that compose the polymeric base of specific films for food packaging applications can migrate into foods during the different phases, in particular during the transport and commercialization of foodstuffs [64]. In this research work, the analysis was performed by using one food simulant, ethanol 50% (*v/v*) (D1), utilized to established the behavior of polymeric films in contact with fatty foods. Table 3 summarizes the migrated levels for all the systems, all the values were lower than the migration limit for foodstuffs contact materials (60 mg kg^−1^ simulant). The obtained results demonstrate the potential applicability of the realized polymeric systems in contact with the food.

Generally, the migrated value increases with increase of QC (or GA) content in the matrix [26,65]. The presence of GA in PVA films induces a slight increase of migration levels in comparison with PVA/QC based films (PVA/5GA = (6.8 ± 0.1) mg kg^−1^ and PVA/10GA = (9.9 ± 0.5) mg kg^−1^ for GA, PVA/5QC = (6.6 ± 0.4) mg kg^−1^ and PVA/10QC = (9.5 ± 0.4) mg kg^−1^ for QC). This behavior can be related to the tendency of GA to better solubilize in aqueous solutions with respect of QC [66] (Table 3). Additionally, the oxidation of foodstuffs during transportation and storage leads to a series of negative changes in food, such as the sensory characteristics of the product (e.g., changes in aesthetic aspect and color, rancidity, and smell) [67,68].

According to this, the efficiency of the produced PVA/GA and PVA/QC based formulations as antioxidant packaging systems was also verified, by testing the reduction of stable free radical DPPH, the obtained data were expressed as RSA (%) and reported in Figure 5c and Table 4. The high antioxidant activity of GA and QC is related to the number and position of the free phenolic hydroxyl groups in the QC and GA molecule [69,70], that intercept free radical chain, inducing the formation of a stable end product, which does not initiate or propagate oxidation of lipids [70].

In Table 4, RSA values for PVA/GA and PVA/QC films evaluated at different times was also presented: no significant differences among the different formulations and among the same formulation at different times were noted (except for the time 0 that shows the lowest value of RSA). The radical scavenging activity reached a maximum reduction of DPPH radicals at 7 days in contact of food simulant (Table 4) (RSA (%): PVA/5GA = (95.0 ± 0.3)%, PVA/10GA = (95.1 ± 0.1)%, PVA/5QC = (96.3 ± 0.2)%, and PVA/10QC = (95.8 ± 0.1)%).

**Figure 6:**
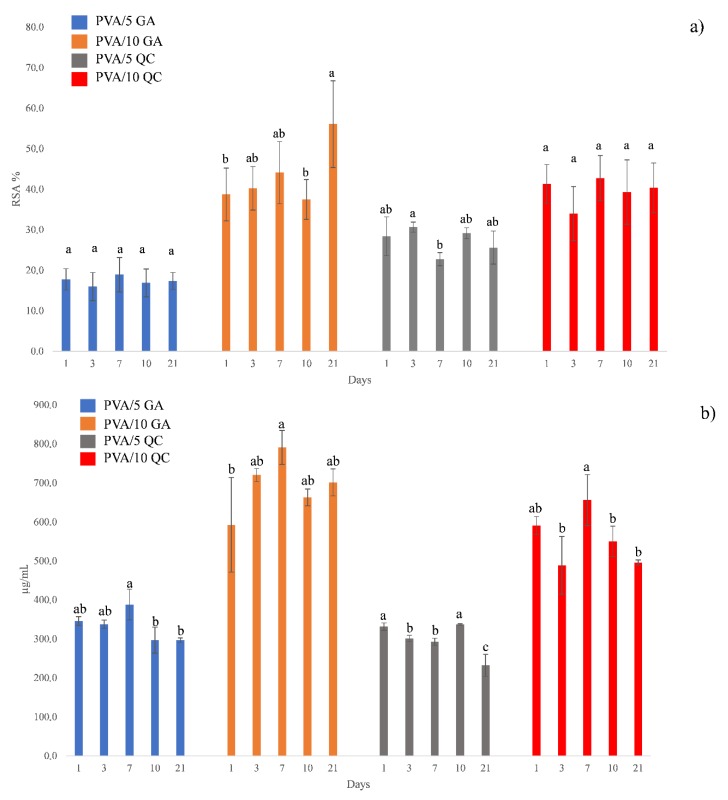
Antioxidant activity (RSA%) of food simulant solutions after specific migration tests from the PVA-based films, evaluated by DPPH assay (**a**) gallic acid (GA) and quercetin (QC) released (μM) from the PVA-based films in food simulant (**b**); Labels indicate significant statistical differences between different time tests for each formulation; where ANOVA was significant the Tukey’s (HSD) test was performed at *p* < 0.05 for the antioxidant activity and at *p* < 0.01 for the release test.

Furthermore, the residual antioxidant activity resulting from specific migration tests of GA and QC containing PVA films was determined (Figure 6a). The measurement of the radical scavenging ability of the food simulant solutions revealed a relevant antioxidant activity detected in all of the samples (RSA% = 56.11 for PVA/10GA, day 21 and RSA% = 40.39 for PVA/10QC, day 21) and even the solutions of films at the lowest concentrations (PVA/5GA and PVA/5QC) exhibited appreciable values of antioxidant capacity (RSA% = 17.39 for PVA/5GA, day 21 and RSA% = 25.65 for PVA/5QC, day 21). Indeed, all samples showed an antioxidant activity in line with the release studies, that will be further discussed.

#### 3.2.5. Release Tests in Food Simulant

Release tests were done according to the European Standard EN 13130-200522 and European Commission Regulation 10/2011 [71], to define the release profile of GA and QC incorporated into the PVA matrix. Thus, PVA/GA and PVA/QC systems were immersed into the simulant used for fatty foods (ethanol 50% *v/v*) for 21 days. All samples were examined at 1, 3, 7, 10, and 21 days, measuring released GA and QC, by UV–vis spectrophotometry (Figure 6b). In all formulations, measured values for released QC and GA are correlated with their concentrations in the films. Indeed, the migration levels from PVA/10GA are increased respect to PVA/5GA, which have been measured, respectively, as 701.54 µg/mL (day 21) and 296.76 µg/mL (day 21). PVA/QC followed the same trend with 495.96 µg/mL (day 21) for PVA/10QC and 231.11 µg/mL (day 21) for PVA/5QC, respectively released from the PVA matrix.

In similar migration experiments, it has been reported that only 1.15% of the QC had been released after 48 h in 95% EtOH and that no more QC was released when the test time was extended to 72 h [24].

FTIR characterization for PVA films after the different release times was also performed, with the main aim of correlating the RSA/release tests information with the physical state of the PVA films containing GA and QC at the two different contents.

**Figure 7:**
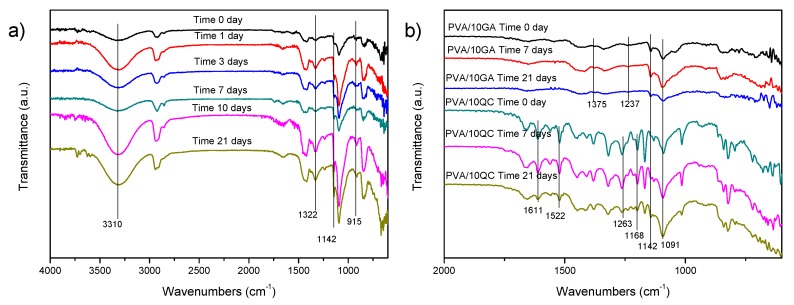
FTIR spectra of neat PVA (**a**), PVA/10GA and PVA/10QC films at the different times of immersion in simulated release solutions (**b**).

In Figure 7a, the spectra of the PVA films at the different times are reported. It can be commented that, in case of neat PVA, no substantial variations were observed, with exception of the peak at 1322 cm^−1^ (due to –CH bending), 1142 cm^−1^ (C–O stretching band), and 915 cm^−1^ (assigned to the CH_2_ rocking vibration) that were more intense, as reported by Zuo et al. [72], PVA membrane after immersion in aqueous ethanol solution showed variation in contact angle values for fully hydrolyzed PVA, attributed to the reorientation of hydroxyl groups at the surface of the membrane. In our case, when the PVA surface contacts the ethanol/water feed mixture, polar −OH groups reoriented at the surface, creating a more hydrophilic conformation (confirmed even by the more intense signal, for our PVA films, of –OH band centered at 3310 cm^−1^ from 0 to 21 days, levelled after 10 days, confirming the results of PVA 99% water contact angle already observed in [72], where ethanol molecules adsorb from solution onto a PVA film surface in an ordered and cooperative way governed by H-bonding, resulting in a more hydrophobic surface, when the hydrolysis degree of PVA is higher than 96%.

**Figure 8:**
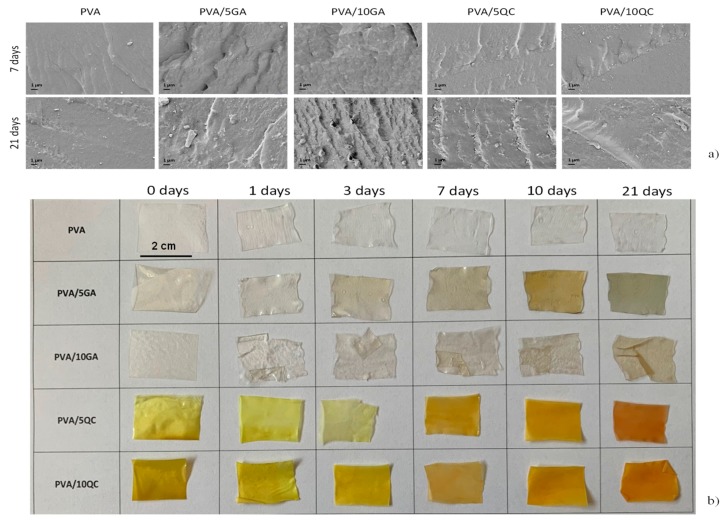
FESEM images of fractured surfaces for PVA based formulation after 7 and 21 days (**a**); visual observation of films of GA and QC based PVA formulations at 0, 1, 3, 7, 10, and 21 days (**b**).

In the case of GA and QC containing films (Figure 7b), spectra analysis evidenced a relative intensity variation for the peaks at 1091 and 1142 cm^−1^, assigned to stretching vibrations of C=O in PVA. More intense peaks at 1375 cm^−1^ and 1237 cm^−1^, related to vibration of phenol alcohol in GA, were noted at 7 and 21 days, while a signal at 1040 cm^−1^ (C–O stretching) resulted less intense: these results are in line with the detected amount of GA in the ethanolic solutions at the same times. In the case of QC, the modified intensity of bands for aromatic C=C at 1611 and 1522 cm^−1^, in-plane bending band of C–H in aromatic hydrocarbon at 1320 cm^−1^, C–O and C–CO–C stretch stretching in the aryl ether ring at 1263 and 1168 cm^−1^ confirmed the evident release of the QC ingredient. On the other hand, it was observed, during the release tests, that the overall swelling degree of GA added films was higher than those of QC added films at 7 and 21 days, and it has been confirmed by analysis of the morphology for swelled fractured surfaces at time 7 and 21 days (Figure 8a): due to the more hydrophilic character of hydroxyl and carboxyl groups containing additive, GA containing films resulted more porous than both neat PVA and PVA/QC films, justifying the increased released amount in case of GA. Nevertheless, as previously observed by Luzi et al. in PVA/Hydroxytyrosol systems [12], the addition of an hydrophilic active ingredient generally enhances the water absorption, which in turn can be also correlated to the lower values of crystallinity degree measured for formulation at 10% wt. of GA, as reported in Table 1.

These interesting packaging materials indeed exhibit a coloration due to the addition of the two ingredients: color of such materials can be considered definitely useful in many applications, such as in the labeling of packaged food, since color of solutions/films can be not only tuned by using natural phytocompounds, but they could be employed as indicators of polymers ageing time when a pH variation is observed. According to this, color parameters for the produced films at time 0 and after different release times were measured. The initial deviation (time 0) of b value from 0.19 ± 0.02 to 5.66 ± 0.02 and increased value of ΔE (6.36 instead of 0.56) showed, for PVA/5GA sample, the tendency towards yellowing and total color changes of films compared to the control (PVA film), essentially due to the presence of well dispersed polyphenolic compounds. When higher amounts of GA was introduced, reduced tendency to yellowness was measured (b = 3.00 ± 0.02), probably related to a limited dispersion of the active ingredient, as already confirmed by mechanical and morphological analysis of PVA/10GA films.

After the release tests (values for 7 and 21 days are also included in Table 5) and immersion in the simulant solution, only the color of the PVA/5GA films deviated towards green (*a* = −3.64 ± 0.08 when compared with neat PVA (*a* = −0.17 ± 0.01)), while the *a* values turned to be less negative (a = −0.65 ± 0.09) in PVA/10GA, and the *b* remained substantially unvaried at 21 days. In the case of QC containing films, even the *a* value was greatly modified towards red, due to the initial color of the filler itself, with yellow basic color shifted to orange-like hues (Figure 8b). Since color change in intelligent films is largely due to the generation of organic acids during anaerobic respiration of anaerobic bacteria or facultative anaerobic bacteria under hypoxic or anaerobic condition, higher acidity values could indicate a time evolving inferior freshness [73]. According to these results, it could be reasonable to use these films as intelligent labels to detect acidity variations in the packages during relatively short food life.

## 4. Conclusions

The results of this study suggest that GA and QC as active ingredients in PVA films can be used to tune not only the antioxidant behavior, but also the deformability of the produced films. It was proved that high content of active ingredients (10% wt.) can be easily released in simulated ethanolic solution and can express high antioxidant activity. At the same time, the active ingredients can hinder the chains packing and inhibit the crystals growth, giving more rigid films, as detected in GA containing films. Nevertheless, QC, that was less dispersible in PVA solutions at the same conditions and showed reduced RSA activity in comparison with GA, can be indeed more efficient in tuning the deformability of the water cast PVA films, due the presence of sole hydroxyl groups carrying agent. The capability of both polyphenols of altering the color of the films after release at different specific times also confirmed the possibility of using these easy processable PVA films as active and intelligent films in food packaging when a specific shelf life (3–21 days) is required.

## Figures and Tables

**Table 1:** Differential scanning calorimetry (DSC) analysis of PVA based films.

Cooling				
Formulations	*T*_g_ (°C)	Δ*H*_c_ (J g^−1^)	*T_c_* (°C)	*X*_c_ (%)
**PVA**	73.1 ± 0.1 ^b^	48.5 ± 2.1 ^a,b^	188.9 ± 0.2 ^a^	30.0 ± 1.3 ^a,b^
**PVA/5GA**	71.9 ± 0.5 ^a,b^	54.0 ± 2.3 ^c^	201.1 ± 0.5 ^c^	33.4 ± 1.3 ^c^
**PVA/10GA**	71.6 ± 0.8 ^a,b^	48.09 ± 0.3 ^a,b^	198.0 ± 2.0 ^b^	29.8 ± 0.2 ^a,b^
**PVA/5QC**	71.5 ± 0.3 ^a^	52.0 ± 0.3 ^b,c^	200.9 ± 0.2 ^c^	32.2 ± 0.2 ^b,c^
**PVA/10QC**	71.1 ± 0.2 ^a^	45.3 ± 2.1 ^a^	191.5 ± 0.9 ^a^	28.1 ± 1.3 ^a^
**Second Heating**				
	***T*_g_ (°C)**	**Δ*H*_m_ (J g^−1^)**	***T_m_* (°C) **	***X*_m_ (%)**
**PVA**	76.6 ± 0.9 ^a^	38.0 ± 3.9 ^a^	217.3 ± 0.1 ^a^	23.5 ± 2.4 ^a^
**PVA/5GA**	82.6 ± 0.1 ^b^	56.7 ± 2.1 ^b^	227.5 ± 0.2 ^c^	35.1 ± 1.3 ^b^
**PVA/10GA**	83.8 ± 1.0 ^b,c^	53.9 ± 2.7 ^b^	223.7 ± 0.6 ^b^	33.3 ± 1.7 ^b^
**PVA/5QC**	84.9 ± 0.3 ^c^	50.9 ± 1.8 ^b^	227.9 ± 0.6 ^c^	31.5 ± 1.2 ^b^
**PVA/10QC**	84.5 ± 0.1 ^b,c^	58.0 ± 3.6 ^b^	224.6 ± 0.5 ^b^	32.281 ± 2.0 ^b^

*T_g_*—glass transition temperature; *T_m_*—melting temperature; *T_c_*—crystallization temperature; Δ*H_m_*—melting enthalpy; Δ*H_c_*—crystallization enthalpy; *X_m_*—melting crystallinity degree (*X_m_*); and *X_c_*—crystallinity degree at cooling scan. ^(a–c)^ Different superscripts within the same column indicate significant differences among formulations regarding the same scan (*p <* 0.05).

**Table 2:** Tensile parameters for PVA based systems.

Formulations	Thickness (Mean Value, μm)	σ_b_ (MPa)	ε_b_ (%)	E (MPa)
**PVA**	45 ± 5 ^b^	67 ± 12 ^a,b^	195 ± 45 ^a,b^	300 ± 80 ^a^
**PVA/5GA**	40 ± 5 ^a^	75 ± 10 ^b^	280 ± 70 ^c^	750 ± 90 ^b^
**PVA/10GA**	50 ± 8 ^c^	55 ± 10 ^a^	190 ± 80 ^a,b^	2000 ± 325 ^c^
**PVA/5QC**	40 ± 7 ^a^	74 ± 10 ^b^	255 ± 35 ^b,c^	305 ± 50 ^a^
**PVA/10QC**	50 ± 7 ^c^	60 ± 10 ^a^	170 ± 10 ^a^	520 ± 110 ^a,b^

^(a–c)^ Different superscripts within the same column indicate significant differences among formulations (*p* < 0.05).

**Table 3:** Overall migration values for different formulations.

Formulations	Overall Migration in Ethanol 50% (*v*/*v*) (mg kg^−1^)
**PVA**	4.1 ± 0.3 ^a^
**PVA/5GA**	6.8 ± 0.1 ^b^
**PVA/10GA**	9.9 ± 0.5 ^c^
**PVA/5QC**	6.6 ± 0.4 ^b^
**PVA/10QC**	9.5 ± 0.4 ^c^

^(a–c)^ Different superscripts within the same column indicate significant differences among formulations (*p* < 0.05).

**Table 4:** Radical scavenging activity (RSA) for PVA based systems.

Formulations	DPPH Scavenging Activity, RSA (%)					
	Time 0 Day	Time 1 Day	Time 3 Days	Time 7 Days	Time 10 Days	Time 21 Days
**PVA/5GA**	90.7 ± 0.1 ^a^	94.3 ± 0.3 ^c^	93.8 ± 0.3 ^c^	95.0 ± 0.3 ^d^	94.1 ± 0.3 ^c^	92.7 ± 0.3 ^b^
**PVA/10GA**	95.3 ± 0.2 ^b^	95.6 ± 0.2 ^b^	95.0 ± 0.3 ^a,b^	95.1 ± 0.1 ^b^	95.6 ± 0.3 ^b^	94.5 ± 0.2 ^a^
**PVA/5QC**	89.0 ± 0.1 ^a^	95.3 ± 0.2 ^c^	96.6 ± 0.3 ^e^	96.3 ± 0.2 ^d,e^	95.7 ± 0.2 ^c,d^	94.3 ± 0.5 ^b^
**PVA/10QC**	91.3 ± 0.2 ^a^	95.5 ± 0.3 ^c^	95.9 ± 02 ^c^	95.8 ± 0.1 ^c^	95.4 ± 0.2^c^	94.5 ± 0.3 ^b^

^(a–e)^ Different superscripts within the same line indicate significant differences among different times evaluated for the same formulation (*p* < 0.05).

**Table 5:** Color coordinates of PVA/GA/QC films.

Formulations	*L* *	*a* *	*b* *	∆*E* *	*Gloss* (°)
**White Control**	99.47 ± 0.00	−0.08 ± 0.01	−0.08 ± 0.01	-	121 ± 0
**PVA**					
**Time 0**	98.98 ± 0.09 ^c^	−0.09 ± 0.00 ^b^	0.19 ± 0.02 ^a^	0.56 ± 0.09 ^a^	252 ± 3 ^b^
**Time 7 days**	98.52 ± 0.01 ^b^	−0.17 ± 0.00 ^a^	0.46 ± 0.03 ^b^	1.09 ± 0.00 ^b^	219 ± 3 ^a^
**Time 21 days**	98.02 ± 0.08 ^a^	−0.17 ± 0.01 ^a^	0.59 ± 0.02 ^c^	1.60 ± 0.06 ^b^	219 ± 6 ^a^
**PVA/5GA**					
**Time 0**	96.77 ± 0.02 ^c^	−0.61 ± 0.00 ^c^	5.66 ± 0.02 ^a^	6.36 ± 0.01 ^a^	234 ± 2 ^b^
**Time 7 days**	91.64 ± 0.17 ^b^	−1.21 ± 0.08 ^b^	12.76 ± 0.15 ^b^	15.08 ± 0.21 ^b^	211 ± 9 ^b^
**Time 21 days**	85.77 ± 0.03 ^a^	−3.64 ± 0.08 ^a^	17.21 ± 0.18 ^c^	22.34 ± 0.17 ^c^	165 ± 7 ^a^
**PVA/10GA**					
**Time 0**	96.98 ± 0.04 ^c^	−0.41 ± 0.05 ^a^	3.00 ± 0.02 ^a^	3.97 ± 0.05 ^a^	164 ± 0 ^b^
**Time 7 day**	95.23 ± 0.01 ^b^	−0.64 ± 0.09 ^a^	6.24 ± 0.06 ^b^	7.63 ± 0.05 ^b^	138 ± 1 ^a^
**Time 21 days**	92.77 ± 0.62 ^a^	−0.65 ± 0.09 ^a^	12.70 ± 0.62 ^c^	14.44 ± 0.82 ^c^	156 ± 10 ^b^
**PVA/5QC**					
**Time 0**	90.84 ± 0.01 ^c^	−9.10 ± 0.13 ^a^	62.73 ± 0.02 ^b^	64.03 ± 0.01 ^b^	52 ± 4 ^b^
**Time 7 days**	83.18 ± 0.01 ^b^	3.23 ± 0.02 ^b^	63.32 ± 0.04 ^c^	65.54 ± 0.04 ^c^	93 ± 2 ^c^
**Time 21 days**	76.91 ± 0.06 ^a^	14.95 ± 0.03 ^c^	50.67 ± 0.23 ^a^	57.23 ± 0.24 ^a^	29 ± 1 ^a^
**PVA/10QC**					
**Time 0**	82.48 ± 0.43 ^b^	−0.29 ± 0.08 ^a^	69.39 ± 0.01 ^c^	71.51 ± 0.10 ^b^	75 ± 3 ^a^
**Time 7 days**	87.04 ± 0.32 ^c^	1.47 ± 0.05 ^b^	50.58 ± 0.92 ^a^	52.19 ± 0.97 ^a^	101 ± 1 ^c^
**Time 21 days**	71.87 ± 0.42 ^a^	20.04 ± 0.39 ^c^	64.30 ± 0.82 ^b^	72.88 ± 0.99 ^b^	95 ± 1 ^b^

^(a–c)^ Different superscripts within the same column indicate significant differences among formulations (*p* < 0.05).
